# Supplementary material for: Integrated bioinformatics analysis of retinal ischemia/reperfusion injury in rats with potential key genes
Source: BMC Genomics. 2024 Apr 15;25:367. doi: 10.1186/s12864-024-10288-0 (PMC11017533; doi:10.1186/s12864-024-10288-0)
Supplement: Supplementary file 34 — Supplementary Material 34. [file 12864_2024_10288_MOESM34_ESM.pdf]

suplementery table 34 GeneMANIA report

Created on : 17 November 2023 15:55:49
Last database update : 13 August 2021 00:00:00
Application version : 3.6.0

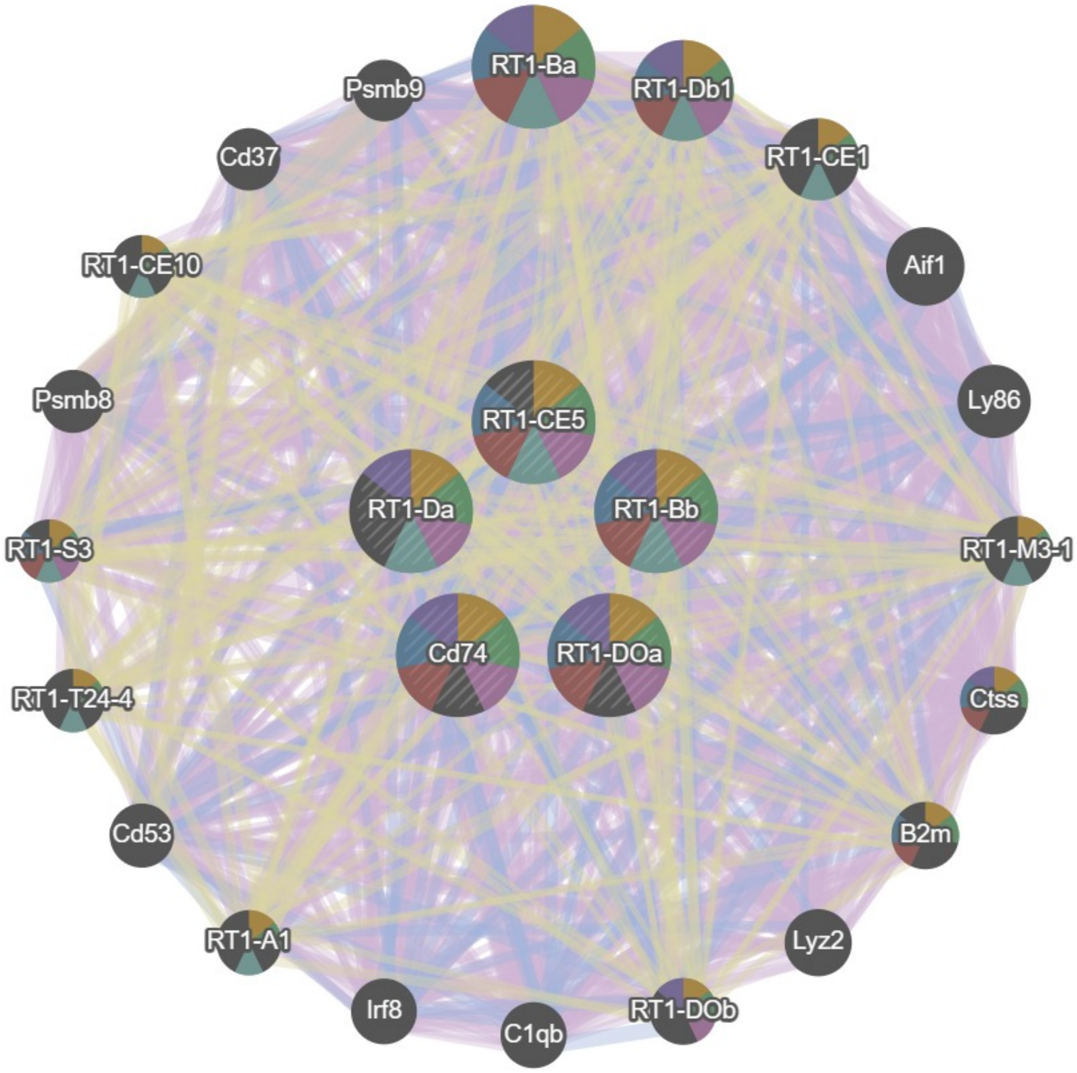

Networks

- Co-expression
- Physical Interactions
- Predicted
- Pathway
- Co-localization
- Shared protein domains

Functions

- antigen processing and presentation of peptide antigen
- antigen processing and presentation
- MHC protein complex
- antigen binding
- antigen processing and presentation of exogenous peptide antigen
- antigen processing and presentation of exogenous antigen
- antigen processing and presentation of peptide antigen via MHC class II

Search parameters

Organism Rattus norvegicus (rat)

Genes RT1-CE5 , Cd74 , RT1-DOa , RT1-Bb , RT1-Da

Network weighting Automatically selected weighting method

Networks

A

Almon-Jusko-2005

B

Bhatnagar-Attie-2014 , BIOGRID-SMALL-SCALE-STUDIES

C

CELL_MAP , Chan-Miron-2007 , Chen-Naus-2012 , Cirilli-Nerlov-2017 , Costigan-Woolf-2010

D

Devarajan-Ketha-Kumar-2012 , Duan-Li-2015

E

Ehyai-McDermott-2018

I

I2D-Bandyopadhyay-Ideker-2010-MAPK-HIGH-Human2Rat , I2D-Bandyopadhyay-Ideker-2010-MAPK-LOW-Human2Rat , I2D-Behrends-Harper-2010-AUTOPHAGY-HIGH-Human2Rat , I2D-Behrends-Harper-2010-AUTOPHAGY-LOW-Human2Rat , I2D-BIND-Fly2Rat , I2D-BIND-Human2Rat , I2D-BIND-Mouse2Rat , I2D-BIND-Worm2Rat , I2D-BIND-Yeast2Rat , I2D-BioGRID-Fly2Rat , I2D-BioGRID-Human2Rat , I2D-BioGRID-Mouse2Rat , I2D-BioGRID-Worm2Rat , I2D-BioGRID-Yeast2Rat , I2D-Formstecher-Daviet-2005-Embryo-Fly2Rat , I2D-Formstecher-Daviet-2005-Head-Fly2Rat , I2D-Giot-Rothbert-2003-High-Fly2Rat , I2D-Giot-Rothbert-2003-Low-Fly2Rat , I2D-HPRD-Human2Rat , I2D-Ingham-Pawson-2005-Human2Rat , I2D-INNATEDB-Human2Rat , I2D-INNATEDB-Mouse2Rat , I2D-IntAct-Fly2Rat , I2D-IntAct-Human2Rat , I2D-IntAct-Mouse2Rat , I2D-IntAct-Worm2Rat , I2D-IntAct-Yeast2Rat , I2D-Jorgensen-Pawson-2009-Human2Rat , I2D-Krogan-Greenblatt-2006-Core-Yeast2Rat , I2D-Krogan-Greenblatt-2006-NonCore-Yeast2Rat , I2D-Li-Vidal-2004-CE-DATA-Worm2Rat , I2D-Li-Vidal-2004-CORE-1-Worm2Rat , I2D-Li-Vidal-2004-CORE-2-Worm2Rat , I2D-Li-Vidal-2004-interolog-Worm2Rat , I2D-Li-Vidal-2004-literature-Worm2Rat , I2D-Li-Vidal-2004-non-core-Worm2Rat , I2D-ManualRat , I2D-MGI-Mouse2Rat , I2D-Miller-Attisano-2009-Human2Rat , I2D-MINT-Fly2Rat , I2D-MINT-Human2Rat , I2D-MINT-Mouse2Rat , I2D-MINT-Worm2Rat , I2D-MINT-Yeast2Rat , I2D-MIPS-Yeast2Rat , I2D-Ptacek-Snyder-2005-Yeast2Rat , I2D-Rual-Vidal-2004-core-Human2Rat , I2D-Rual-Vidal-2004-non-core-Human2Rat , I2D-Stanyon-Finley-2004-

I

CellCycle-Fly2Rat , I2D-Stelzl-Wanker-2005-High-Human2Rat , I2D-Tarassov-PCA-Yeast2Rat , I2D-vonMering-Bork-2002-High-Yeast2Rat , I2D-vonMering-Bork-2002-Low-Yeast2Rat , I2D-vonMering-Bork-2002-Medium-Yeast2Rat , I2D-Wang-Orkin-2006-EScmplxlow-Mouse2Rat , I2D-Wu-Li-2007-Human2Rat , I2D-Yu-Vidal-2008-GoldStd-Yeast2Rat , INTERPRO , IREF-bhf-ucl , IREF-bind , IREF-bind-translation , IREF-biogrid , IREF-corum , IREF-dip , IREF-hpidb , IREF-intact , IREF-intcomplex , IREF-matrixdb , IREF-mbinfo , IREF-mint , IREF-mppi , IREF-quickgo , IREF-SMALL-SCALE-STUDIES , IREF-uniprotpp

J

Jin-Zhang-2007 , Jolly-Estrem-2005

K

Kimpel-McBride-2007

L

López-Boado-Churg-2010

M

Marin-Kuan-Schilter-2006 , Martins-Marques-Girão-2015 , Martins-Rebelo-2018 , Mertz-Peng-2015 , Meyer-Meyer-2004 , Moon-Kim-2014

N

Na-Peng-2012

P

PFAM , Piazzi-Cocco-2013 , Popiolek-Pausch-2011

R

Rimbault-Galibert-2009 , Roth-Suter-2011 , Römer-Zell-2014 B

S

Stevenson-Butler-2007 , Strøm-Sheikh-2004

T

Thomas-Bowyer-2010

V

von Kriegsheim-Kolch-2009

W

Walker-Bilbe-2004 , Wang-You-2006 , Wilson-Koh-2005 , Wisler-Vonderfecht-2011

Y

Yang-Paschen-2012 , Yang-Salminen-2012 , Young-Tannock-2012

Genes

Gene	Description	Rank
RT1-CE5	RT1 class I, locus CE5 [Source:RGD Symbol;Acc:1595868]	N/A
RT1-Bb	RT1 class II, locus Bb [Source:RGD Symbol;Acc:3469]	N/A
RT1-DOa	RT1 class II, locus DOa [Source:RGD Symbol;Acc:3477]	N/A
Cd74	CD74 molecule [Source:RGD Symbol;Acc:2313]	N/A
RT1-Da	RT1 class II, locus Da [Source:RGD Symbol;Acc:1593283]	N/A
RT1-Ba	RT1 class II, locus Ba [Source:RGD Symbol;Acc:1595867]	1
RT1-Db1	RT1 class II, locus Db1 [Source:RGD Symbol;Acc:1593282]	2
RT1-CE1	RT1 class I, locus1 [Source:RGD Symbol;Acc:1595869]	3
Aif1	allograft inflammatory factor 1 [Source:RGD Symbol;Acc:61924]	4
Ly86	lymphocyte antigen 86 [Source:RGD Symbol;Acc:1309936]	5
RT1-M3-1	RT1 class Ib, locus M3, gene 1 [Source:RGD Symbol;Acc:3494]	6
Ctss	cathepsin S [Source:RGD Symbol;Acc:621513]	7
B2m	beta-2 microglobulin [Source:RGD Symbol;Acc:2189]	8
Lyz2	lysozyme 2 [Source:RGD Symbol;Acc:3026]	9
RT1-DOb	RT1 class II, locus DOb [Source:RGD Symbol;Acc:1595814]	10
C1qb	complement C1q B chain [Source:RGD Symbol;Acc:2229]	11
Irf8	interferon regulatory factor 8 [Source:RGD Symbol;Acc:1312024]	12
RT1-A1	RT1 class Ia, locus A1 [Source:RGD Symbol;Acc:1595924]	13
Cd53	Cd53 molecule [Source:RGD Symbol;Acc:2310]	14
RT1-T24-4	RT1 class I, locus T24, gene 4 [Source:RGD Symbol;Acc:1598233]	15
RT1-S3	RT1 class Ib, locus S3 [Source:RGD Symbol;Acc:735037]	16
Psmb8	proteasome 20S subunit beta 8 [Source:RGD Symbol;Acc:3426]	17
RT1-CE10	RT1 class I, locus CE10 [Source:RGD Symbol;Acc:1359292]	18
Cd37	CD37 molecule [Source:RGD Symbol;Acc:62035]	19
Psmb9	proteasome 20S subunit beta 9 [Source:RGD Symbol;Acc:3427]	20

Networks

Co-expression 39.02%

Wang-You-2006 2.67%

Organ-specific expression profiles of rat mammary gland, liver, and lung tissues treated with targretin, 9-cis retinoic acid, and 4-hydroxyphenylretinamide. Wang et al (2006). *Mol Cancer Ther*

Co-expression with 138,363 interactions from GEO

Stevenson-Butler-2007 2.57%

Comprehensive gene expression profiling of rat lung reveals distinct acute and chronic responses to cigarette smoke inhalation. Stevenson et al (2007). *Am J Physiol Lung Cell Mol Physiol*

Co-expression with 149,476 interactions from GEO

Römer-Zell-2014 B 2.32%

Cross-platform toxicogenomics for the prediction of non-genotoxic hepatocarcinogenesis in rat. Römer et al (2014). *PLoS One*

Co-expression with 342,316 interactions from GEO

Rimbault-Galibert-2009 2.14%

RNA profiles of rat olfactory epithelia: individual and age related variations. Rimbault et al (2009). *BMC Genomics*

Co-expression with 250,164 interactions from GEO

Wisler-Vonderfecht-2011 2.05%

Raf inhibition causes extensive multiple tissue hyperplasia and urinary bladder neoplasia in the rat. Wisler et al (2011). *Toxicol Pathol*

Co-expression with 362,068 interactions from GEO

Yang-Salminen-2012 2.02%

Identification of urinary microRNA profiles in rats that may diagnose hepatotoxicity. Yang et al (2012). *Toxicol Sci*

Co-expression with 290,575 interactions from GEO

Chan-Miron-2007 2.01%

Serial transplantation of NMU-induced rat mammary tumors: a model of human breast cancer progression. Chan et al (2007). *Int J Cancer*

Co-expression with 239,920 interactions from GEO

Young-Tannock-2012 2.00%

Changes in bowel microbiota induced by feeding weanlings resistant starch stimulate transcriptomic and physiological responses. Young et al (2012). *Appl Environ Microbiol*

Co-expression with 247,577 interactions from GEO

Duan-Li-2015 1.98%

Transcriptome analyses reveal molecular mechanisms underlying functional recovery after spinal cord injury. Duan et al (2015). *Proc Natl Acad Sci U S A*

Co-expression with 406,614 interactions from GEO

Almon-Jusko-2005 1.94%

Temporal profiling of the transcriptional basis for the development of corticosteroid-induced insulin resistance in rat muscle. Almon et al (2005). *J Endocrinol*

Co-expression with 126,978 interactions from GEO

Strøm-Sheikh-2004 1.91%

Co-expression 39.02%

Strøm-Sheikh-2004

Identification of a core set of genes that signifies pathways underlying cardiac hypertrophy. Strøm et al (2004). *Comp Funct Genomics*

Co-expression with 119,202 interactions from GEO

López-Boado-Churg-2010 1.88%

Modification of the rat airway explant transcriptome by cigarette smoke. López-Boado et al (2010). *Inhal Toxicol*

Co-expression with 328,336 interactions from GEO

Jolly-Estrem-2005 1.87%

Pooling samples within microarray studies: a comparative analysis of rat liver transcription response to prototypical toxicants. Jolly et al (2005). *Physiol Genomics*

Co-expression with 138,955 interactions from GEO

Costigan-Woolf-2010 1.86%

Multiple chronic pain states are associated with a common amino acid-changing allele in KCNS1. Costigan et al (2010). *Brain*

Co-expression with 141,130 interactions from GEO

Roth-Suter-2011 1.80%

Gene expression-based in vivo and in vitro prediction of liver toxicity allows compound selection at an early stage of drug development. Roth et al (2011). *J Biochem Mol Toxicol*

Co-expression with 111,402 interactions from GEO

Meyer-Meyer-2004 1.69%

Altered mRNA expression of genes related to nerve cell activity in the fracture callus of older rats: A randomized, controlled, microarray study. Meyer et al (2004). *BMC Musculoskelet Disord*

Co-expression with 139,512 interactions from GEO

Wilson-Koh-2005 1.65%

Microarray analysis of postictal transcriptional regulation of neuropeptides. Wilson et al (2005). *J Mol Neurosci*

Co-expression with 135,074 interactions from GEO

Marin-Kuan-Schilter-2006 1.65%

A toxicogenomics approach to identify new plausible epigenetic mechanisms of ochratoxin a carcinogenicity in rat. Marin-Kuan et al (2006). *Toxicol Sci*

Co-expression with 136,806 interactions from GEO

Thomas-Bowyer-2010 1.61%

Endoplasmic reticulum stress responses differ in meninges and associated vasculature, striatum, and parietal cortex after a neurotoxic amphetamine exposure. Thomas et al (2010). *Synapse*

Co-expression with 254,504 interactions from GEO

Kimpel-McBride-2007 1.40%

Functional gene expression differences between inbred alcohol-preferring and -non-preferring rats in five brain regions. Kimpel et al (2007). *Alcohol*

Co-expression with 124,453 interactions from GEO

Physical Interactions 25.61%

von Kriegsheim-Kolch-2009 4.02%

Cell fate decisions are specified by the dynamic ERK interactome. von Kriegsheim et al (2009). *Nat Cell Biol*

Physical Interactions with 219 interactions from iRefIndex

Physical Interactions 25.61%

Jin-Zhang-2007 3.76%

Identification of novel proteins associated with both alpha-synuclein and DJ-1. Jin et al (2007). *Mol Cell Proteomics*

Physical Interactions with 126 interactions from iRefIndex

Martins-Marques-Girão-2015 2.16%

Interacting Network of the Gap Junction (GJ) Protein Connexin43 (Cx43) is Modulated by Ischemia and Reperfusion in the Heart. Martins-Marques et al (2015). *Mol Cell Proteomics*

Physical Interactions with 178 interactions from iRefIndex

IREF-intcomplex 1.73%

Physical Interactions with 33 interactions from iRefIndex

Ehyai-McDermott-2018 1.51%

FMRP recruitment of -catenin to the translation pre-initiation complex represses translation. Ehyai et al (2018). *EMBO Rep*

Physical Interactions with 130 interactions from BioGRID

IREF-quickgo 1.43%

Physical Interactions with 602 interactions from iRefIndex

IREF-dip 1.11%

Physical Interactions with 255 interactions from iRefIndex

Chen-Naus-2012 1.10%

Association of connexin43 with E3 ubiquitin ligase TRIM21 reveals a mechanism for gap junction phosphodegron control. Chen et al (2012). *J Proteome Res*

Physical Interactions with 103 interactions from BioGRID

Moon-Kim-2014 1.06%

Interactome analysis of AMP-activated protein kinase (AMPK)- 1 and - 1 in INS-1 pancreatic beta-cells by affinity purification-mass spectrometry. Moon et al (2014). *Sci Rep*

Physical Interactions with 435 interactions from BioGRID

IREF-mbinfo 0.93%

Physical Interactions with 17 interactions from iRefIndex

BIOGRID-SMALL-SCALE-STUDIES 0.88%

Physical Interactions with 3,299 interactions from BioGRID

Devarajan-Ketha-Kumar-2012 0.82%

The sclerostin-bone protein interactome. Devarajan-Ketha et al (2012). *Biochem Biophys Res Commun*

Physical Interactions with 96 interactions from BioGRID

IREF-bind-translation 0.78%

Physical Interactions with 549 interactions from iRefIndex

Piazzi-Cocco-2013 0.62%

Phosphoinositide-specific phospholipase C 1b (PI-PLC 1b) interactome: affinity purification-mass spectrometry analysis of PI-PLC 1b with nuclear protein. Piazzi et al (2013). *Mol Cell Proteomics*

Physical Interactions with 132 interactions from iRefIndex

IREF-corum 0.55%

Physical Interactions with 109 interactions from iRefIndex

Physical Interactions 25.61%

IREF-intact 0.53%

Physical Interactions with 1,415 interactions from iRefIndex

Bhatnagar-Attie-2014 0.51%

Phosphorylation and degradation of tomosyn-2 de-represses insulin secretion. Bhatnagar et al (2014). *J Biol Chem*

Physical Interactions with 201 interactions from BioGRID

IREF-bind 0.46%

Physical Interactions with 335 interactions from iRefIndex

IREF-matrixdb 0.41%

Physical Interactions with 121 interactions from iRefIndex

IREF-mppi 0.41%

Physical Interactions with 86 interactions from iRefIndex

IREF-biogrid 0.32%

Physical Interactions with 3,694 interactions from iRefIndex

IREF-mint 0.27%

Physical Interactions with 796 interactions from iRefIndex

Cirilli-Nerlov-2017 0.12%

Insights into specificity, redundancy and new cellular functions of C/EBPa and C/EBPb transcription factors through interactome network analysis. Cirilli et al (2017). *Biochim Biophys Acta Gen Subj*

Physical Interactions with 106 interactions from BioGRID

IREF-uniprotpp 0.10%

Physical Interactions with 149 interactions from iRefIndex

Predicted 21.99%

I2D-ManualRat 4.08%

Predicted with 7 interactions from I2D

I2D-Miller-Attisano-2009-Human2Rat 2.68%

Application of an integrated physical and functional screening approach to identify inhibitors of the Wnt pathway. Miller et al (2009). *Mol Syst Biol*

Predicted with 16 interactions from I2D

I2D-vonMering-Bork-2002-High-Yeast2Rat 1.46%

Comparative assessment of large-scale data sets of protein-protein interactions. von Mering et al (2002). *Nature*

Predicted with 81 interactions from I2D

I2D-Wu-Li-2007-Human2Rat 1.37%

Systematic identification of SH3 domain-mediated human protein-protein interactions by peptide array target screening. Wu et al (2007). *Proteomics*

Predicted with 57 interactions from I2D

I2D-INNATEDB-Mouse2Rat 1.25%

InnateDB: facilitating systems-level analyses of the mammalian innate immune response. Lynn et al (2008). *Mol Syst Biol*

Predicted with 268 interactions from I2D

Predicted 21.99%

I2D-BioGRID-Mouse2Rat 1.07%

BioGRID: a general repository for interaction datasets. Stark et al (2006). *Nucleic Acids Res*

Predicted with 1,003 interactions from I2D

I2D-Li-Vidal-2004-interolog-Worm2Rat 0.99%

A map of the interactome network of the metazoan C. elegans. Li et al (2004). *Science*

Predicted with 56 interactions from I2D

I2D-HPRD-Human2Rat 0.87%

Development of human protein reference database as an initial platform for approaching systems biology in humans. Peri et al (2003). *Genome Res*

Predicted with 4,002 interactions from I2D

I2D-BioGRID-Yeast2Rat 0.83%

BioGRID: a general repository for interaction datasets. Stark et al (2006). *Nucleic Acids Res*

Predicted with 2,556 interactions from I2D

I2D-INNATEDB-Human2Rat 0.81%

InnateDB: facilitating systems-level analyses of the mammalian innate immune response. Lynn et al (2008). *Mol Syst Biol*

Predicted with 1,411 interactions from I2D

I2D-BioGRID-Human2Rat 0.69%

BioGRID: a general repository for interaction datasets. Stark et al (2006). *Nucleic Acids Res*

Predicted with 16,098 interactions from I2D

I2D-IntAct-Mouse2Rat 0.66%

The IntAct molecular interaction database in 2010. Aranda et al (2010). *Nucleic Acids Res*

Predicted with 2,741 interactions from I2D

I2D-Jorgensen-Pawson-2009-Human2Rat 0.63%

Cell-specific information processing in segregating populations of Eph receptor ephrin-expressing cells. Jørgensen et al (2009). *Science*

Predicted with 127 interactions from I2D

I2D-vonMering-Bork-2002-Medium-Yeast2Rat 0.48%

Comparative assessment of large-scale data sets of protein-protein interactions. von Mering et al (2002). *Nature*

Predicted with 212 interactions from I2D

I2D-BioGRID-Worm2Rat 0.47%

BioGRID: a general repository for interaction datasets. Stark et al (2006). *Nucleic Acids Res*

Predicted with 128 interactions from I2D

I2D-BIND-Human2Rat 0.45%

BIND--a data specification for storing and describing biomolecular interactions, molecular complexes and pathways. Bader et al (2000). *Bioinformatics*

Predicted with 534 interactions from I2D

I2D-Behrends-Harper-2010-AUTOPHAGY-LOW-Human2Rat 0.44%

Network organization of the human autophagy system. Behrends et al (2010). *Nature*

Predicted with 2,501 interactions from I2D

I2D-MGI-Mouse2Rat 0.44%

Predicted 21.99%

I2D-MGI-Mouse2Rat

Ontological visualization of protein-protein interactions. Drabkin et al (2005). *BMC Bioinformatics*

Predicted with 99 interactions from I2D

I2D-Yu-Vidal-2008-GoldStd-Yeast2Rat 0.43%

High-quality binary protein interaction map of the yeast interactome network. Yu et al (2008). *Science*

Predicted with 33 interactions from I2D

I2D-BioGRID-Fly2Rat 0.42%

BioGRID: a general repository for interaction datasets. Stark et al (2006). *Nucleic Acids Res*

Predicted with 1,327 interactions from I2D

I2D-Bandyopadhyay-Ideker-2010-MAPK-LOW-Human2Rat 0.28%

A human MAP kinase interactome. Bandyopadhyay et al (2010). *Nat Methods*

Predicted with 212 interactions from I2D

I2D-BIND-Mouse2Rat 0.27%

BIND--a data specification for storing and describing biomolecular interactions, molecular complexes and pathways. Bader et al (2000). *Bioinformatics*

Predicted with 138 interactions from I2D

I2D-Ingham-Pawson-2005-Human2Rat 0.20%

WW domains provide a platform for the assembly of multiprotein networks. Ingham et al (2005). *Mol Cell Biol*

Predicted with 17 interactions from I2D

I2D-Bandyopadhyay-Ideker-2010-MAPK-HIGH-Human2Rat 0.16%

A human MAP kinase interactome. Bandyopadhyay et al (2010). *Nat Methods*

Predicted with 83 interactions from I2D

I2D-MIPS-Yeast2Rat 0.14%

MPact: the MIPS protein interaction resource on yeast. Güldener et al (2006). *Nucleic Acids Res*

Predicted with 63 interactions from I2D

I2D-Krogan-Greenblatt-2006-NonCore-Yeast2Rat 0.14%

Global landscape of protein complexes in the yeast Saccharomyces cerevisiae. Krogan et al (2006). *Nature*

Predicted with 79 interactions from I2D

I2D-MINT-Mouse2Rat 0.11%

MINT: a Molecular INTeraction database. Zanzoni et al (2002). *FEBS Lett*

Predicted with 186 interactions from I2D

I2D-IntAct-Human2Rat 0.10%

The IntAct molecular interaction database in 2010. Aranda et al (2010). *Nucleic Acids Res*

Predicted with 8,515 interactions from I2D

I2D-IntAct-Yeast2Rat 0.10%

The IntAct molecular interaction database in 2010. Aranda et al (2010). *Nucleic Acids Res*

Predicted with 955 interactions from I2D

Pathway 8.15%

CELL_MAP 8.15%

Pathway 8.15%

CELL_MAP

Pathway with 9 interactions from Pathway Commons

Co-localization 2.86%

Walker-Bilbe-2004 2.86%

Applications of a rat multiple tissue gene expression data set. Walker et al (2004). *Genome Res*

Co-localization with 193,336 interactions from GEO

Shared protein domains 2.37%

INTERPRO 1.49%

Shared protein domains with 567,125 interactions from InterPro

PFAM 0.88%

Shared protein domains with 529,507 interactions from Pfam
