# Supplementary figures and images for: Integrated bioinformatics analysis of retinal ischemia/reperfusion injury in rats with potential key genes
Source: BMC Genomics. 2024 Apr 15;25:367. doi: 10.1186/s12864-024-10288-0 (PMC11017533; doi:10.1186/s12864-024-10288-0)

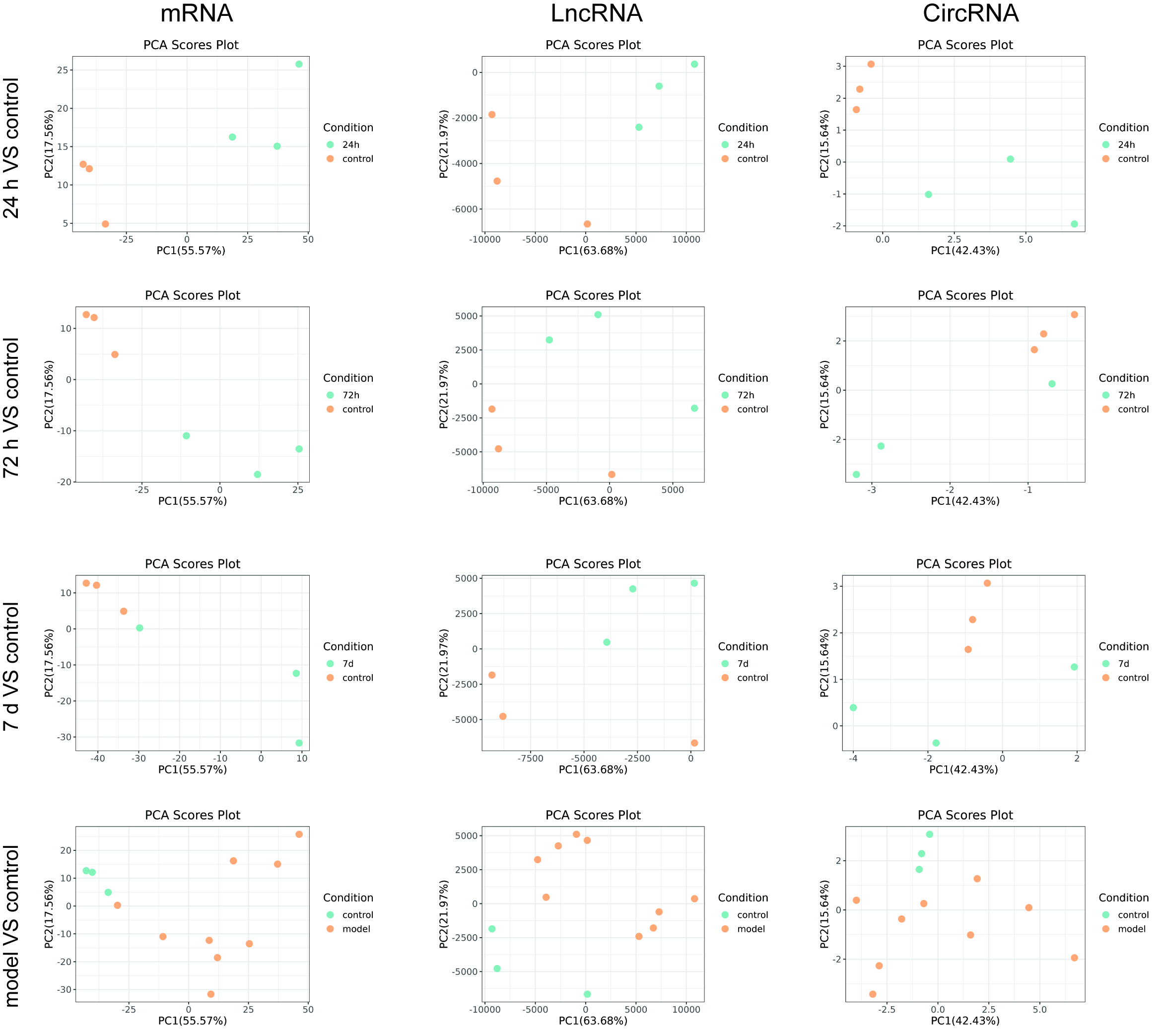

Supplement: Supplementary file 91 — Supplementary Material 91. [file 12864_2024_10288_MOESM91_ESM.tif]

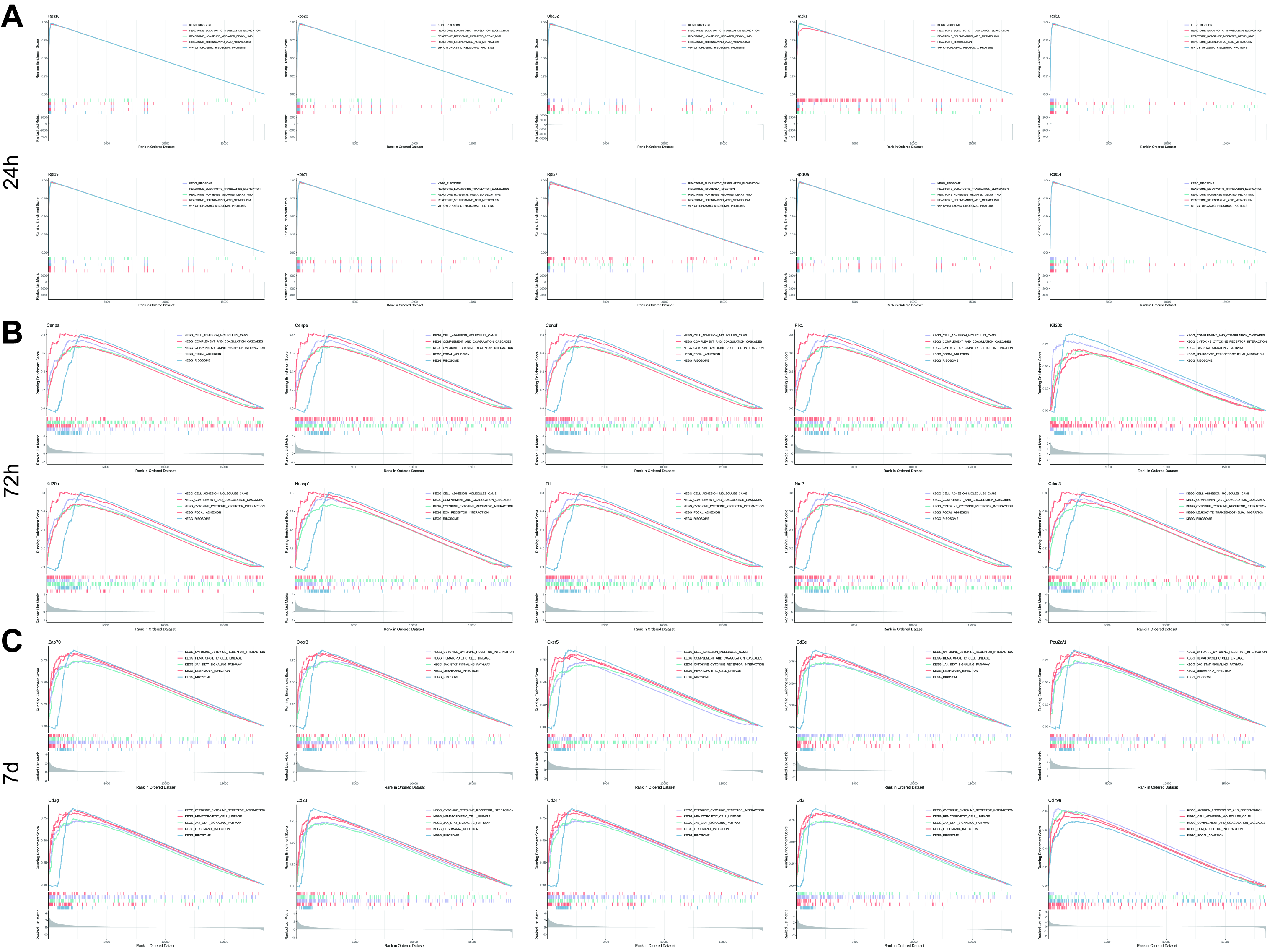

Supplement: Supplementary file 92 — Supplementary Material 92. [file 12864_2024_10288_MOESM92_ESM.tif]
